# Prediction of function in daily life following multidisciplinary rehabilitation for individuals with chronic musculoskeletal pain; a prospective study

**DOI:** 10.1186/1471-2474-8-65

**Published:** 2007-07-10

**Authors:** Monica Lillefjell, Steinar Krokstad, Geir Arild Espnes

**Affiliations:** 1 Department of Social Work and Health Science, Norwegian University of Science and Technology, Trondheim, Norway; 2 The Nord-Trøndelag Health Study Research centre, Norwegian University of Science and Technology, Trondheim, Norway; 3 Department of Occupational Therapy, Sør-Trøndelag University College, Trondheim, Norway; 4 Department of Nursing, Sør-Trøndelag University College, Trondheim, Norway

## Abstract

**Background:**

The prevalence of chronic musculoskeletal pain is high, with widespread negative economic, psychological, and social consequences for the individual. It is therefore important to find ways to predict the outcome of rehabilitation programmes in terms of function in daily life. The aims of this study were to investigate the improvements over time from multidisciplinary rehabilitation in terms of pain and function, and analyse the relative impact of individual and psychosocial factors as predictors of function in daily life in individuals with chronic musculoskeletal pain.

**Methods:**

A prospective study was conducted among one hundred and forty three (*N *= 143) musculoskeletal pain patients. Measures of pain, function, and functional health status were obtained at baseline, after 5 weeks of intensive training, at the end of the 57-week rehabilitation programme, and at a 1 year follow-up, using validated self-administrated measures. Linear regression analysis was applied to investigate the relative impact of musculoskeletal pain, individual-, and psychosocial factors in function.

**Results:**

The participants studied showed a significant increase in function during the 57 weeks rehabilitation period. There was also a significant increase in function from the end of the rehabilitation period (57th week) to the one year follow-up measures. Pain intensity associated significantly with pain experience over all measurement periods. High levels of pain intensity (*β *= .42**) and pain experience (*β *= .37*), and poor psychological capacity (*β *= -.68*) at baseline, as well as poor physiological capacity (*β *= -.44**) and high levels of anxiety (*β *= .48**) and depression (*β *= .58***) at the end of the rehabilitation program were the most important prognostic factors of variance in functioning over the 4 measurement periods.

**Conclusion:**

The data suggest that physical capacity, emotional distress and coping skills should be priority areas in rehabilitation programmes to improve functioning in daily life.

## Background

Chronic musculoskeletal pain represents an important cause of reduced function in daily life, and constitutes a significant and increasing medical, social, and economic challenge in industrialized countries [1,2]. In more than 90 % of the musculoskeletal pain cases, no organic reason can explain the pain that for some individuals persists and gets worst to the point where it considerably limits function in everyday activities [3-5]. General pain, viewed as a multidimensional phenomenon with varying degrees of severity, distribution and functional impact, is considered to be chronic if it lasts for more than three months [3,6,7]. Chronification is not only tied to the duration of pain. Chronic pain is found to be associated with a multitude of secondary stressors such as sleep disruption, unemployment and interpersonal tensions [3,8,9], and psychosocial factors are considered to be among the most important variables that influence the total health picture. The influence of individual and psychosocial factors in function is moreover believed to be stronger for people with chronic musculoskeletal pain [6,10]. Pain and function can also be approached in a cultural and historical context, and are viewed as multidimensional phenomena that are influenced by many factors, such as the effect of previous experience and cultural beliefs, as well as sensory input [7,10]. In accordance to the International Association for the Study of Pain (IASP), the experience of pain is connected to emotions and is defined as 'an unpleasant sensory and emotional experience associated with actual or potential tissue damage, or described in terms of such damage' [[11], p.108]. A study by Rudy, Lieber, Boston, Gourley and Baysal [12] concluded that more than 90 % of the variance in performance among disabled individuals with chronic musculoskeletal pain was predicted by psychosocial factors; self-efficacy, perceived emotional and physical functioning, pain intensity, and pain cognition being the most important. This is supported by Geisser, Robinson and Miller [10] maintaining that individual and psychosocial factors were deemed to be of great importance in the experience of pain. The consequences of pain for a person's everyday life are therefore not only dependent on the underlying pathophysiological impairments, but to a large extent decided by that person's perception of the disease in their present life situation. Depression, reported to be highly prevalent among people with chronic pain [2,13], can take many forms and vary in the number and severity of symptoms. Even milder symptoms of depression have been found to influence the experience of pain. Both somatic and cognitive symptoms of depression are associated with perceived psychosocial functioning among people with chronic musculoskeletal pain, even when controlling for pain intensity and other measures [2,10]. Pain-related anxiety, the belief that pain is a sign of damage or harm to the body, and that activities that might cause pain should be avoided are also believed to be important contributors to disability and adjustment among people with chronic pain [10,14]. Therefore, the individual's understanding of the symptoms and the impact of the symptoms on everyday life might be an important way of understanding pain and function.

Chronic widespread pain and poor health functioning are significantly associated with a number of environmental factors [15-17], acting both through and independently of disease. This is emphasized by Krokstad and Westin [18] who demonstrate the importance and impact of social-, non-medical-, and contextual determinants in disability. Factors such as little social support, little social anchorage, or little need of being social are found to significantly increase the odds for a person to experience a high level of pain [19,20]. The development of widespread chronic pain is also found to be predicted by higher age, drinking alcohol weekly, smoking, traumas in childhood and a family history of chronic pain. However, optimistic attitudes about how the pain will interfere with daily life, the individual's social interaction, and the individual's ability to receive assistance are factors that are found to predict pain reduction [9,19,21-24]. Multidisciplinary treatments, in general, are found to effectively improve the functioning of chronic musculoskeletal pain patients in daily life. Such treatments are more cost-effective than alternative pain control treatments (i.e. 'conservative' care and surgery), and achieve equal or greater efficiency [25,26]. People who have completed treatment typically report decreased pain intensity, less depression and less pain related anxiety, improved levels of pain coping skills, and increased function in daily life.

Chronic pain in the musculoskeletal system and responses to rehabilitation treatment has often been studied in terms of clinical factors and objective determinants of the person [9,10,19,22]. Results from several studies indicate that physical-, psychological-, and socioeconomic variables play a major role in how pain is experienced, as well as how individuals respond to rehabilitation treatment for chronic musculoskeletal pain conditions [9,10,18-21]. While advanced designs are appearing more frequently in chronic musculoskeletal pain research, there is a need for prospective, inception studies so that we can learn more about the nature of the risk factors being studied. Longitudinal follow-up studies, conducted in a real clinical setting, are therefore still needed.

The present study uses a biopsychosocial theoretical approach and the empirical findings discussed [27-29] in order to: 1. Examine improvement in function over time in individuals with chronic musculoskeletal pain participating in a multidisciplinary rehabilitation programme, and 2. Analyse the relative impact of individual and psychosocial factors as predictors of pain intensity, pain experience, and function in daily life in individuals with chronic musculoskeletal pain participating in a multidisciplinary rehabilitation programme.

## Methods

### Subjects

The study sample consisted of 143 (*N *= 143) individuals, aged 20–67 (mean age = 45.7/SD = 8.9), with chronic (> 3 month) musculoskeletal pain, who participated in a 57-week long multidisciplinary rehabilitation programme at a rehabilitation centre in central-Norway. Data were collected at four points in time; at the start of the rehabilitation, after 5 weeks of intensive training, at the end of the 57-week rehabilitation period, and at a 1 year follow-up after end of the rehabilitation period. All participants (*N *= 143) completed the 57 weeks rehabilitation period, however, the follow-up response 1 year after the participants completed the rehabilitation period was 51 % (*n *= 72). The majority of the participants (*N *= 143) were women (74 %), and 79 % of the participants reported to have primary or technical/vocational school for 1–2 years. In addition, the majority of the participants reported to be unskilled or skilled workers/craftspeople (59 %). In order to compare the characteristics of the study sample with the general population in the same geographic area, data were used from an age-matched group (*n *= 52186, mean age = 43/*SD *= 12.7) from the Nord-Trøndelag Health Study (The HUNT 2 Study). The study was approved by Norwegian Social Science Data Service (NSD) and the Regional Medical Ethical Committee of Mid-Norway (REK). All patients were volunteers and gave their informed consent. Confidentiality was emphasized.

### Treatment program

The multidisciplinary rehabilitation programme (see Table 1), based on a biopsychosocial theoretical model [29,30], consisted of a 5-week intensive period, where the participants attended approximately 6 h/day, 4 days a week, and a follow-up period of 52 weeks, where the participants attended approximately 6 h/day, 1–3 days a week. The participants were assigned to the rehabilitation programme by their medical doctor based on interviews, observations, and clinical tests. Formulation of individual training and exercise programmes is based on the mapping of the participants. All participants had a personal supervisor, and individual counselling is offered during the training period.

**Table 1:** Content of the multidisciplinary rehabilitation programme

Period	Intervention	Duration
Period I: Mapping of the participants resources/intensive training period	• Introduction to the rehabilitation programme • Mapping physical-, psychological-, and social function • Individual counselling-based on the mapping; preparation of a long-term plan for the rehabilitation process in cooperation with their medical doctor, social security office and the employer. • Individual and group-based training to improve functional capacity: 1. Individual exercise programme with focus on e.g., endurance, strength, mobility, and relaxation techniques, 2. Group-based education/training in different health related subjects e.g., body structure, diet, exercise planning, coping strategies, communication, strategies for conflict negotiations, and social security system 3. Indoor and outdoor activities every day	*6 h/day, 4 days a week in 5 weeks*
Period II: Follow-up training/rehabilitation period	Functional capacity training continues (individual and group-based, indoor/outdoor activities, education), individual counselling, clarifying function and work ability, prepare a plan for work re-entry in cooperation with the employer, for example.	*6 h/day, 1–3 days a week in 52 weeks*
During/after finishing the rehabilitation period	In addition to the regular rehabilitation programme (57 weeks), the rehabilitation centre offers exercise groups e.g., endurance groups, water activity groups, and relaxation training groups in the participant's local community.	*1 h/1–3 days a week*

In cooperation with the National Health Insurance Office, Employment office, employer and other Public Health Services, an individual tailored education and coping process was emphasized that sought to increase functional capacity, decrease affective distress, and educate patients about the positive health process. Although all patients did not receive exactly the same standardized intervention, as would be expected in a randomized controlled trial, our aim with this study was to examine individual effects in function in real clinical settings.

### Instruments and procedures

Self-reporting measures were administrated individually to the participants at the rehabilitation centre. Data were collected at baseline, after the 5-week intensive period, after the 57-week rehabilitation period and at the one year follow-up after the participants finished the rehabilitation period.

The Visual Analogue Scale (VAS) [31,32] was employed to assess variables on pain (worst imaginable pain, how troublesome the pain is), physical capacity (muscle strength, endurance capacity, energy, mobility, and balance), psychological capacity (good feeling inside, mood, feeling valuable, extroverted/introverted, optimistic/pessimistic, calm, and balanced), coping (feeling of coping in daily life, control and influence in daily life), and cognitive capacity (concentration, memory, understand/evaluate information, and knowledge). The VAS is a line of 10 cm on which pain marks are scored in millimetres, representing the continuum of the symptom to be rated. Instructions about how to rate the present pain, how troublesome the pain is and the present function/capacity were given along with the scale. VAS variables were used as independent variables (predictors of outcome). Moreover, pain intensity and pain experience were used as outcome measures as well. The use of the VAS is well established in chronic pain populations, and test-retest reliability of the scale has been satisfactory with a reproducibility of 0.75–0.83 [31,33]. The scale has also been used in creative ways to further explore the phenomenon of pain perception and reporting, in addition to explore other health-related phenomena [31,34,35]. Factor analysis (varimax method) [36], extracted with eigenvalues > 1.00 as a criterion, indicated that items could be grouped according to the intended constructs presented above.

Function in daily life was measured using the Norwegian version of the Functional Health Status measurement COOP/WONCA Charts [37]. The COOP/WONCA charts, used as an outcome indicator (dependent), measure six core aspects of functional status: physical fitness, feelings, daily activities, social activities, changes in health and overall health. Each item is rated on a five-point ordinal scale ranging from 1 ('no limitation at all') to 5 ('severely limited'). The test-retest reliability of the original Dartmouth version and the Norwegian version was found satisfactory (r = 0.74–0.86) [38]. The charts of function and feelings have been reported to correlate well with other measures of physical and emotional functioning respectively, such as the Barthel Index and the Zung Depression Scale [32].

Anxiety and depression, used as predictors (independent) of function (functional health status, pain intensity and pain experience), were assessed by using the Hospital Anxiety and Depression Scale (HADS) [32,39]. HADS is a brief assessment of anxiety and depression, consisting of 14 items divided into two sub-scales for anxiety and depression, in which the patient rates each item on a four-point scale. Individual items are scored from 0–3 to 3-0, depending on the direction of the wording of the items. The scores of the items represent the degree of distress: none = 0, unbearably = 3. Tests for reliability (test-retest) of the scale have been satisfactory with a reproducibility of 0.67–0.77 [32,39]. Factor analysis (varimax method) [36], extracted with eigenvalues > 1.00 as a criterion, indicated that items could be grouped according to the two main constructs.

The participants' self-reporting about education level, type of job, financial matters, social network, sleep disturbance, tiredness, and history of childhood trauma (independent variables) was supplemented by personal interviews. The self-reporting of traumas include experiences such as; bullying, physical-, emotional-, and/or sexual abuse. Except for education, categorized in four levels, all information retrieved from the interviews was categorized in two levels of categorical variables. The internal consistency was acceptable in this study and measures such as Cronbach's alpha coefficients were calculated at 0.80–0.85.

### Statistical analysis

Data were analysed using SPSS for Windows (version 14.0) software. Frequencies, percentages, mean values and standard deviation were calculated for continuous and categorical variables. Multivariate tests (single group repeated measures design) [36] of the significance of the repeated-measures effect (Pillai's Trace) were provided in order to examine the long-term improvements (variance due to passage of time) of the multidisciplinary rehabilitation programme in terms of functional health status (COOP/WONCA), pain intensity (VAS), pain experience (VAS), anxiety (HADS), and depression (HADS). T-tests were used to compare the sample (*N *= 143) with the HUNT population from the same geographical area, on the anxiety and depression variables. For the initial selection of potential determinants for the outcome measures pain intensity, pain experience and functional health status (physical fitness, feelings, daily activities, social activities and overall health), univariate linear regression analysis, done on the baseline, were used with of significance level of p < 0.05. Subsequently, all independent variables that showed significant associations with the outcome measures (dependent) were considered for inclusion into the multivariate linear regression models. These analyses were carried out separately for the definition of outcome variables (pain intensity, pain experience, functional health status: physical fitness, feelings, daily activities, social activities and overall health). In order to identify which variables predict change over time best, all measurements across the rehabilitation period (3 times) were included in steps in the same model with effects of these variables on the estimated change of the outcome variables (functional health status, pain intensity, pain experience) over the 3 measurement periods (T1-T3). The dependent variable at Time 1 was entered first in the model to control for its effect. In addition, all measurements across all times were included in steps in the same model with effects of these variables on the estimated change of the outcome variables (functional health status, pain intensity, pain experience) over the 4 measurement periods (T1-T4). To control for the effect of the dependent variable at T1, the variable was entered first in the model. In the final multivariate models only variables with p-value less than 0.05 were retained. A *p*-value of less than 0.05 was considered statistically significant.

## Results

### Response and baseline characterization of the sample

All patients (*N *= 143) included completed the 57-week rehabilitation programme, which gave a response of 100 % at the end of the rehabilitation period. However, the response percentage decreased to 51 % (*n *= 72) at the 1 year follow-up questionnaire on pain intensity, pain experience, and functional health status. The non-response group reported mean pain and pain experience measured by VAS at respectively 75.4 and 68.0 at the end of the rehabilitation period. Further the mean measures on functional health status (COOP/WONCA charts) were calculated at: physical fitness; 2.87, feelings; 2.73, daily activities; 3.08, social activities; 2.23, and overall health; 3.08 at the end of the rehabilitation period in the non-response group. Back, shoulders, and neck were the most common pain locations in the sample, and 93.8 % of the participants reported pain in more than two locations. As seen in Table 2, the majority (68 %) of the sample was married, and the total per cent exposed to traumas in childhood in the present pain sample was 37 %. Sixty nine per cent reported sleeplessness and 74 % reported tiredness in everyday daily life. By comparison, the age-matched population from the same geographic area (HUNT 2) (aged 20–67) consisted of 47.5 % men and 52.5 % women, 70 % reported to have basic or secondary education, and 22.5 % of the HUNT population reported to be unskilled or skilled workers/craftspeople. The portion reporting poor social network in the chronic musculoskeletal pain sample was equal to the HUNT population (Table 2).

**Table 2:** Characteristics of present sample (*N *= 143) at baseline compared to the HUNT population (*n *= 52 186).

Characteristics	*N *= 143	*n *= 52 186
Married/cohabitant (%)	71.3	71.7
Smoking (%)	53.8	36.6
Traumas in childhood (%)^a^	37.1	-
Sleeplessness (%)	69.9	31.3
Tiredness (%)	74.8	45.5
Poor Social network (%)	17.5	17.5
Poor Economy (%)	34.3	15.5

^a^Not measured in the HUNT population

### Functional status

**Figure 1:**
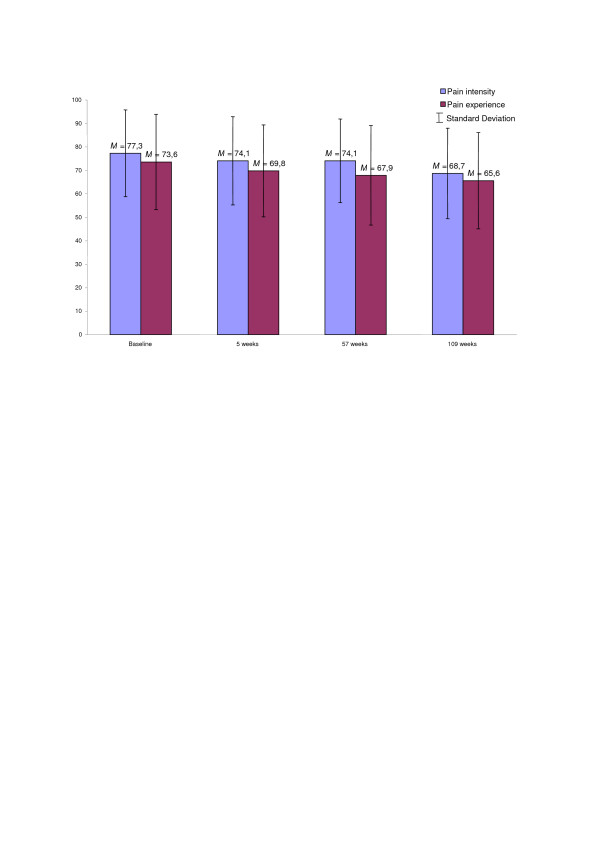
**Mean and standard deviation of pain intensity and pain experience measured by VAS**. Mean (*M*) and standard deviation (*SD*) of pain intensity and pain experience (how troublesome the pain is) measured by Visual Analogue Scale (0–100) at the start of the rehabilitation period, after 5 weeks of intensive training, at the end of the 57-week rehabilitation period (*N *= 143), and at the one year follow-up measures (*n *= 72) in present musculoskeletal pain sample.

Figure 1 shows the mean and standard deviations of pain intensity and pain experience (how troublesome the pain is) measured at 3 points in times during the rehabilitation period and at a one year follow-up. Pain intensity and pain experience significantly (*p *< 0.01) decreased from the start of the rehabilitation period to the one year follow-up measures at 109 weeks. Table 3 show the long-term improvements (trend over time) of the multidisciplinary rehabilitation programme with a significant improvement in cognitive- (*p *< 0.001), physiological-, (*p *< 0.001), and psychological (*p *< 0.01) capacity, measured by VAS, in the pain sample during the 57-week rehabilitation period. In addition, scores on the Hospital Anxiety and Depression Scale (HADS), seen in Table 3, showed a significant (*p *< 0.01) reduction in both anxiety and depression during the rehabilitation period. Despite this reduction, the present pain sample still scored significantly (*p *< 0.001) higher on the anxiety and depression variables at all measurement points during the rehabilitation period compared to the HUNT population.

**Table 3:** Multivariate tests of the significance for the repeated-measures effect on functional status in the present sample (Pillai's Trace *V*)

	Baseline	5 weeks	57 weeks				
	
Variables (*N *= 143).	*M(SD)*	*M(SD)*	*M(SD)*	*V*	*F*	*df*	*p*
VAS							
Physiological capacity^a^	39.9(15.6)	43.6(15.6)	45.8(18.1)	.14	11.61	2	000***
Psychological capacity^b^	58.8(16.4)	59.8(16.0)	62.8(18.3)	.07	5.97	2	.003**
Coping capacity^c^	57.4(15.9)	56.8(16.2)	57.3(19.2)	.00	.12	2	.884
Cognitive capacity^d^	47.9(20.1)	50.0(17.5)	54.3(19.1)	.13	10.81	2	.000***
HAD							
Anxiety	8.83(4.29)	8.65(4.46)	7.93(4.53)	.06	5.16	2	.007**
Depression	6.03(4.16)	5.59(3.96)	5.08(4.30)	.07	5.34	2	.006**

^a^Physiological; muscle strength, endurance capacity, energy, mobility and balance, ^b^Psychological; good feeling inside, mood, feeling valuable, extroverted/introverted, optimistic/pessimistic, calm and balanced, ^c^Coping; feeling of not coping in daily life, control and influence in daily life, ^d^Cognitive; concentration, memory, understand/evaluate information, knowledge. *p < 0.05. **p < 0.01. ***p < 0.001.

**Figure 2:**
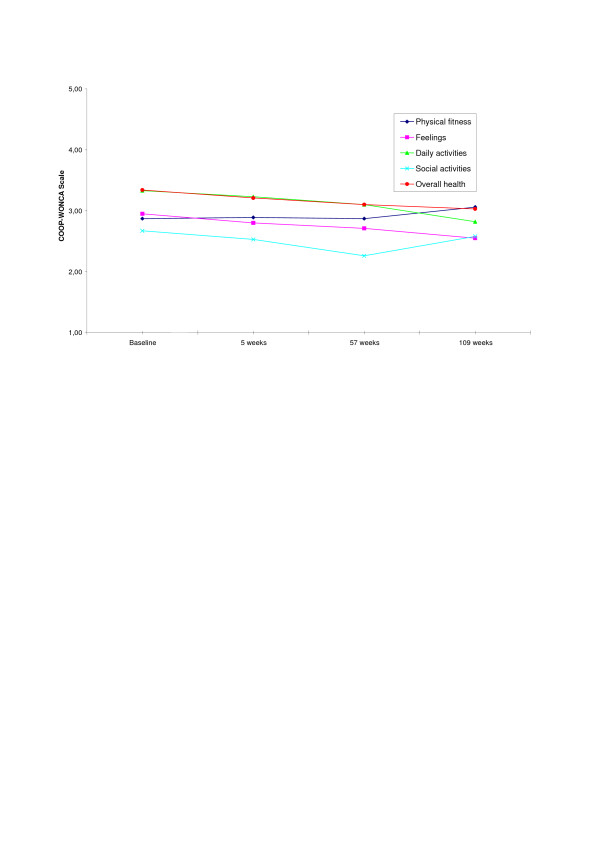
**Repeated measures mean for Functional Health Status (COOP-WONCA) in the present musculoskeletal pain sample**. Mean Functional Health status measured at the start of the rehabilitation period, after 5 weeks of intensive training, at the end of the 57 weeks rehabilitation period (*N *= 143), and at the one year follow-up measures (*n *= 72). 1 = no limitation at all, 5 = severely limited.

In Figure 2, measures of function in daily life using the COOP/WONCA charts (Functional Health Status) are presented as mean values. Functional health status significantly increased on the variables feelings (*p *< 0.05), daily activities (*p *< 0.05), social activities (*p *< 0.001), and overall health (*p *< 0.01) from baseline to the end of the 57th week of the rehabilitation period in present sample. However, a comparison of present musculoskeletal pain sample with a normative randomized sample (*N *= 2864) from the Ullensaker study [38] on the COOP/WONCA charts, demonstrates that the musculoskeletal pain sample (*N *= 143) still report significantly lower function (*p *< 0.01) on all core aspects of functional health status at the end of the 57-week rehabilitation period. A relative low response (51 %) might limit the relevance of the one year follow-up analysis, however the follow-up measures (109 weeks) on functional health status showed that the participants continued to improve their function in daily activities (*M *= 2.82/*SD *= .95), feelings (*M *= 2.55/*SD *= 1.27), and overall health (*M *= 3.03/*SD *= .77), compared to the 57th week measures (daily activities *M *= 3.10/*SD *= .95, feelings *M *= 2.71/*SD *= 1.15, and overall health *M *= 3.10/*SD *= .85). However, the improvement in function was significant only in daily activities (*p *< 0.05). In addition, the participants reported a decrease in physical fitness and social activities one year after they completed the rehabilitation period, compared to the 57th week measures. The decrease in physical fitness and social activities was not significant, however.

### Univariate linear regression analysis

Univariate linear regression analysis, done on the baseline, showed that a multitude of potential prognostic indicators associated significantly with our primary outcome measure functional health status (physical fitness, feelings, daily activities, social activities and overall health).

Here, poor physiological capacity (*F *= 19.92/*p *< 0.000) and high pain experience (*F *= 4.06/*p *< 0.046) significantly associated with poor physical fitness, while limitation on the outcome variable feelings associated with poor financial situation (*F *= 13.05/*p *< 0.000), experience of traumas in childhood (*F *= 11.37/*p *< 0.001), poor social network (*F *= 9.85/*p *< 0.002) and high levels of anxiety (*F *= 111.61/*p *< 0.000) and depression (*F *= 66.42/*p *< 0.000). Moreover, there was a significant association between limitation in feelings and poor physiological (*F *= 5.77/*p *< 0.018)-, psychological (*F *= 68.93/*p *< 0.000)-, coping (*F *= 12.59/*p *< 0.001)-, and cognitive (*F *= 13.97/*p *< 0.000) capacity in present musculoskeletal pain sample.

Sleeplessness (*F *= 6.73/*p *< 0.010), high levels of pain intensity (*F *= 18.26/*p *< 0.000), pain experience (*F *= 25.27/*p *< 0.000), anxiety (*F *= 6.58/*p *< 0.011) and depression (*F *= 6.52/*p *< 0.012) were in the univariate analysis significantly associated with limitation in daily activities. Furthermore, limitation in daily activities associated with poor physiological (*F *= 11.90/*p *< 0.001)-, psychological (*F *= 5.10/*p *< 0.025) -, coping (*F *= 6.89/*p *< 0.010)-, and cognitive (*F *= 5.03/*p *< 0.026) capacity. Limitation in social activities was significantly associated with poor social network (*F *= 4.93/*p *< 0.028), high levels of anxiety (*F *= 12.27/*p *< 0.001) and depression (*F *= 10.41/*p *< 0.002), and reporting poor physiological (*F *= 10.41/*p *< 0.002)-, psychological (*F *= 22.16/*p *< 0.000) -, coping (*F *= 7.79/*p *< 0.006)-, and cognitive (*F *= 10.57/*p *< 0.001) capacity in present pain sample.

The univariate analysis, done on the baseline, also showed a significant association between poor overall health and high age (*F *= 4.53/*p *< 0.035), experience of traumas in childhood (*F *= 7.68/*p *< 0.006), poor social network (*F *= 10.62/*p *< 0.001), and high levels of pain intensity (*F *= 7.73/*p *< 0.006). In addition, limitation in overall health was significantly associated with high levels of anxiety (*F *= 10.81/*p *< 0.001) and depression (*F *= 24.40/*p *< 0.000), and poor physiological (*F *= 24.77/*p *< 0.000)-, psychological (*F *= 16.19/*p *< 0.000) -, coping (*F *= 17.04/*p *< 0.000)-, and cognitive (*F *= 11.19/*p *< 0.001) capacity in present musculoskeletal pain sample.

Poor physiological capacity was the only variable that significantly associated with high levels of pain intensity (*F *= 7.88/*p *< 0.006) and pain experience (*F *= 11.39/*p *< 0.001) in the univariate analysis, done on the baseline, in the musculoskeletal pain sample.

### Multivariate linear regression analysis

Table 4 summarizes the multivariate linear regression analysis with effects of the independent variables (only significant variables included in the table) across the rehabilitation period (3 times) on the estimated change of the outcome (functional health status) over the 3 measurement periods (T1-T3). Cognitive capacity (*β *= -.17*) was the only baseline (T1) measure that associated significantly with functional health status (overall health) in the final model (Table 4). Poor physiological (*β *= -.24*/-.45***) (T2 and T3)- and psychological (*β *= -.38**) (T3) capacity, high levels of anxiety (*β *= .59***) and depression (*β *= .31*) (T3), as well as high levels of pain intensity (*β *= .15*) and pain experience (*β *= .35**) (T3), were the strongest predictors of variance of functioning (functional health status measured by COOP/WONCA) over the 3 measurement periods (57 week rehabilitation period) (Table 4).

**Table 4:** Effects of independent variables on the estimated change of functional health status over the 3 measurement periods

Variables (*N *= 143).	*B (SE)*	*β*	Δ*R*^2^	*R*^2^
Physical fitness				
Step 1: Dependent variable T-1	-	-	.23	.23
Step 2: Independent variables T-1	-	-	.08	.32
Step 3: Independent variables T2-3	-	-	.10	.42
Physiological capacity T3	-.07(.00)	-.30**	-	-
Feelings				
Step 1: Dependent variable T-1	-	-	.20	.20
Step 2: Independent variables T-1	-	-	.11	.32
Step 3: Independent variables T2-3	-	-	.40	.72
Psychological capacity T3	-.02(.00)	-.38**	-	-
Anxiety T3	.15(.02)	.59***	-	-
Daily activities				
Step 1: Dependent variable T-1	-	-	.15	.15
Step 2: Independent variables T-1	-	-	.08	.23
Step 3: Independent variables T2-3	-	-	.28	.52
Pain experience T3	.01(.00)	.35**	-	-
Social activities				
Step 1: Dependent variable T-1	-	-	.12	.12
Step 2: Independent variables T-1	-	-	.07	.20
Step 3: Independent variables T2-3	-	-	.23	.44
Depression T3	.07(.03)	.31*	-	-
Overall health				
Step 1: Dependent variable T-1	-	-	.20	.20
Step 2: Independent variables T-1	-	-	.12	.33
Cognitive capacity	-.00(.00)	-.17*	-	-
Step 3: Independent variables T2-3	-	-	.30	.64
Physiological capacity T2	-.01(.00)	-.24*	-	-
Physiological capacity T3	-.02(.00)	-.45***	-	-
Pain intensity T3	.00(.00)	.15*	-	-

*B *= Unstandardized Coefficients, *SE *= Std. Error, *β *= Standardized Coefficients derived from the final step. Δ*R*^2 ^= change in explanation rate in each step. *R*^2 ^= proportion of variance explained. *p < 0.05. **p < 0.01. ***p < 0.001.

Linear regression analysis (*B *= Unstandardized Coefficients, *SE *= Std. Error, *β *= Standardized Coefficients derived from the final step) was also performed on all measurements across all times with effects of the independent variables on the estimated change of functional health status over the 4 measurement periods (not included in table). Variance in functioning (functional health status measured by COOP/WONCA) over the 4 measurement periods (T1-T4) were significantly predicted by experience of traumas in childhood (*B*(*SE*) = .50(.24), *β *= .29*), high levels of pain intensity (*B*(*SE*) = .02(.00), *β *= .42**) and pain experience (*B*(*SE*) = .02(.00), *β *= .37*), and poor psychological capacity (*B*(*SE*) = .05(.02), *β *= -.68*) at baseline (T1). Moreover, poor physiological capacity (*B*(*SE*) = -.02(.00), *β *= -.44**) and high levels of anxiety (*B*(*SE*) = .13(.04), *β *= .48**) and depression (*B*(*SE*) = .16(.04), *β *= .58***) at the end of the rehabilitation program (T3) were found to significantly predict the variance in functioning (functional health status measured by COOP/WONCA) over the 4 measurement periods.

Variance in pain intensity over the 3 measurement periods (not included in table) was significantly associated with high levels of pain experience (T3) (*B*(*SE*) = .54(.07), *β *= .65***) and physiological capacity (T3) (*B*(*SE*) = .19(.09), *β *= .19*). The association between pain intensity and physiological capacity was however not significant over the 4 measurements periods. High levels of pain intensity (T3) (*B(SE) *= .58(.08), *β *= .48***) was the strongest predictor of variance in pain experience over the 3 measurement periods. Moreover, the association between pain intensity (T3) (*B*(*SE*) = .48(.22), *β *= .36*) and pain experience was significant over all 4 measurement periods.

## Discussion

This study showed that a multitude of factors had an effect on pain intensity, pain experience, and functional health status over the measurement periods in a Norwegian sample, and different variables affected different aspects of daily life function. The participants were found to significantly improve several aspects related to function during the rehabilitation period. However, it still might be relevant to question in what way these changes influence the everyday life of the people in this sample. Ultimately, the consequences of chronic musculoskeletal pain for everyday function depend not only on pain intensity and pain experience, but also on the individual and on each person's unique set of earlier experiences, values, and environmental conditions. This illustrates the complexity of chronic pain conditions, where the person's perception of pain and function and his/her experiences of what it means in their everyday life might be an important way of understanding the complexity. Therefore, the relative influence of psychosocial factors on function may vary a lot depending on the activity the individuals are engaged in [40]. For the person that receives treatment the importance of the overall effectiveness of the rehabilitation programme is re-establishing function. However, the programme is also important from a broader perspective. The reduction in pain intensity and pain experience along with improved function in daily life indicate a positive effect from the extensive rehabilitation programme. This is further underlined by the increase in function in daily activities, feelings, and overall health from the 57th week of the rehabilitation period to one year after the participants finished the rehabilitation programme. However, it is important to note that although the participants improved function during the rehabilitation period, they still report significantly lower function on all core aspects of functional health status compared to a normative sample from the Ullensaker study (*N *= 2864) [38] at all points of measurement. In addition, the significant decrease in self-reported physical fitness and social activities from the 57th week of the rehabilitation period to one year after the participants finished the rehabilitation programme give rise for concern. Lack of physical fitness and participation in social activities might later on influence several aspects of function in daily life and might not be beneficial to the individuals or to the society. Future studies should therefore try to clarify the long-term effect of multidisciplinary rehabilitation programmes for individuals with chronic musculoskeletal pain in terms of function in daily life. Non-specific musculoskeletal pain is an increasing health problem in the Norwegian population. The increased study of individual rehabilitation in a formal rehabilitation programme must not reduce focus on primary prevention programmes at a population level and on the social- and economic policy implications of the present findings.

Several studies [5,10,41] suggest that the impairment of function in daily life is associated with several psychosocial factors. The intent of this study was to study the long-term improvements of a multidisciplinary rehabilitation programme, by focusing on interactions and the influence of a broad range of socio-demographic and psychosocial factors in pain intensity, pain experience, and functional health status. In order to do that, the predictors of change in pain intensity, pain experience, and functional health status over time were studied. The relationship between emotional distress, chronic pain and function in daily life has been shown before [13]. In this study experience of traumas in childhood, emotional distress, high levels of pain intensity and pain experience, and poor physical capacity, measured at baseline, were significantly predicting lack of improvement in functional health status over all measurement periods. In terms of emotional distress, it is also relevant to notice the relative high percentage (37 %) of traumas in the present pain sample. The participants report significantly higher levels of anxiety and depression before, during, and after the treatment period compared to the normative population from the same geographical area (The HUNT Study). Taken together, and supported by previous studies as well [2,10,13], this illustrates the complexity and the relative importance of emotional distress in chronic musculoskeletal pain conditions.

A study by Palermo and Kiska [42] suggested that sleep disturbance is closely linked to mood disturbance. However, less is known about the complex interrelationship between emotional distress, sleeplessness and function in daily life among adults with chronic musculoskeletal pain conditions. More than 69 % of our sample reported sleep disturbance in daily life. However, sleeplessness adjusted for emotional distress like anxiety and depression, was not found to be useful in the prediction of function. The results in this study confirm the physical capacity and coping aspects in multidisciplinary rehabilitation found in past research as well [43-45], suggesting that physical exercise, behavioral and cognitive-behavioral treatment for chronic pain reduces pain, pain distress, and improves daily functioning. Moreover, in accordance with a study by Lame, Peters, Vlaeyen, Kleef and Patijn [46], our study indicates the relevance of pain experience in predicting function, and that function in daily life might be associated with beliefs about pain.

The participants in this study are not randomly sampled; they represent all patients participating in the rehabilitation programme at a given period. The drop-out rate on long term follow-up might limit the power of the follow-up analysis and results. However, the participants are representative for people with chronic musculoskeletal pain seeking help at a rehabilitation clinic with respect to age, sex, pain conditions, working ability and sick leave. The sample and the general population from the same geographical area are almost identical with regard to age distribution, family situation, social network, and education level. This allows scrutinization of differences between the sample and the general population without taking the factors mentioned above into consideration as an explanatory variable. Even with a 100 % response at the end of the rehabilitation period, a relatively small number of participants could lead to a reduction in the power of the analysis and decrease the possibility of generalization. Another limitation of the results is the possibility of bias related to the self-reported data [47]. However, multidimensional rehabilitation, as in present study, represents an approach that has the potential to effectively focus on function in daily life, not necessarily on rendering the individual symptom free, which might provide potentially greater reliability in the self-assessment of function. Some might possibly argue that improvement over time is not very surprising since pain patients often are selected close to their worse status. The participants in present study are long-term chronic pain patients, in some cases reporting pain duration of more than 10 years. Due to the chronicity considerable improvements in function may therefore not be anticipated. The VAS scale has been used in creative ways to explore the phenomenon of pain perception and reporting in addition to exploring other health related phenomena [32,34,35]. However, the VAS measures used in present study should be further validated. Despite several shortcomings, the study highlights important perspectives in a real clinical setting that should be taken into account in rehabilitation of chronic musculoskeletal pain, and in primary prevention at a population level.

## Conclusion

This study has evaluated a complexity of factors that have theoretical or empirical relationships to function in daily life in a sample with persisting musculoskeletal pain. The results of this study highlight important individual perspectives in chronic musculoskeletal pain. These results are important to better understand which variables are most useful in helping patients re-establish function during a rehabilitation programme and they show how to address the variables that affect the outcome. In a broader perspective, and as seen in relation to the high prevalence of people with chronic musculoskeletal pain conditions, it is also important to pay attention to the underlying causes of incidence and primary prevention at a population level.

## Competing interests

The author(s) declare that they have no competing interests.

## Authors' contributions

ML, SK and GAE designed the study, ML collected the data, ML analysed and wrote up the manuscript. ML, SK, GAE revised the manuscript. All authors read and approved the final manuscript.

## Pre-publication history

The pre-publication history for this paper can be accessed here:


